# Prediction of Lung Function in Adolescence Using Epigenetic Aging: A Machine Learning Approach

**DOI:** 10.3390/mps3040077

**Published:** 2020-11-09

**Authors:** Md Adnan Arefeen, Sumaiya Tabassum Nimi, M. Sohel Rahman, S. Hasan Arshad, John W. Holloway, Faisal I. Rezwan

**Affiliations:** 1 Department of Computer Science Electrical Engineering, University of Missouri-Kansas City, Kansas City, MO 64110, USA; aa4cy@mail.umkc.edu (M.A.A.); snvb8@mail.umkc.edu (S.T.N.); 2 Department of Computer Science and Engineering, Bangladesh University of Engineering and Technology, Dhaka 1205, Bangladesh; msrahman@cse.buet.ac.bd; 3 Clinical and Experimental Sciences, Faculty of Medicine, University of Southampton, Southampton SO16 6YD, UK; S.H.Arshad@soton.ac.uk; 4 The David Hide Asthma and Allergy Research Centre, St Mary’s Hospital, Newport, Isle of Wight PO30 5TG, UK; 5 Human Development and Health, Faculty of Medicine, University of Southampton, Southampton SO16 6YD, UK; J.W.Holloway@soton.ac.uk; 6 School of Water, Energy and Environment, Cranfield University, Cranfield MK43 0AL, UK

**Keywords:** lung function, epigenetic aging, machine learning, feature selection, hyperparameter tuning

## Abstract

Epigenetic aging has been found to be associated with a number of phenotypes and diseases. A few studies have investigated its effect on lung function in relatively older people. However, this effect has not been explored in the younger population. This study examines whether lung function in adolescence can be predicted with epigenetic age accelerations (AAs) using machine learning techniques. DNA methylation based AAs were estimated in 326 matched samples at two time points (at 10 years and 18 years) from the Isle of Wight Birth Cohort. Five machine learning regression models (linear, lasso, ridge, elastic net, and Bayesian ridge) were used to predict FEV_1_ (forced expiratory volume in one second) and FVC (forced vital capacity) at 18 years from feature selected predictor variables (based on mutual information) and AA changes between the two time points. The best models were ridge regression (R^2^ = 75.21% ± 7.42%; RMSE = 0.3768 ± 0.0653) and elastic net regression (R^2^ = 75.38% ± 6.98%; RMSE = 0.445 ± 0.069) for FEV_1_ and FVC, respectively. This study suggests that the application of machine learning in conjunction with tracking changes in AA over the life span can be beneficial to assess the lung health in adolescence.

## 1. Introduction

In recent years, the concept of biological aging, as opposed to chronological aging, has gained considerable popularity in understanding the aging process due to its stronger relation with phenotypes and diseases [1]. DNA methylation (DNAm), an epigenetic process, can provide biomarkers to estimate biological aging, known as “epigenetic aging”. There are several methods available to estimate epigenetic aging [2,3,4,5,6], and among them, the Horvath method for epigenetic age estimation (DNAmAge) is used widely and has shown high accuracy, with an average correlation > 0.90 with chronological age [4]. Age acceleration (AA) is the difference between epigenetic age and chronological age, and both DNAmAge and AA are highly correlated with chronological age. However, another epigenetic age acceleration measure calculated from the residuals of regression (AA_res_), between epigenetic and chronological ages, is not correlated with chronological age and is thought to represent true biological effects on age related phenotypes. In addition, another related measure is the intrinsic epigenetic age acceleration (IEAA), which is independent of age related changes of the cellular composition of blood [7]. Several recent studies, using the Horvath method, have found that age acceleration is associated with a number of diseases and phenotypes, such as obesity [8], Alzheimer’s disease [9], Down’s syndrome [10], Huntington disease [11], HIV [12], Parkinson’s disease [13], earlier menopause [14], and overall mortality [15]. Studies have also shown that lung function can be influenced by epigenetic age accelerations as quantified in peripheral blood DNAm [16,17].

Lung development is a continuous process from childhood to adolescence [18]. Low adult lung function can be the result of poor growth in childhood, which may cause excessive decline in adult life [19], and it has been found, in many studies, that children with poor lung function also experience reduced lung function in adulthood [20,21,22,23,24]. While lung function is dependent on age, gender, height, and ethnicity [18], it can be influenced by both genetics [25] and environmental exposure [26,27,28]. Studies have shown that DNAm, measured in peripheral blood, is associated with lung functions [29]. Changes in DNAm from childhood to adolescence have been found to be associated with lung function during adolescence in females [30]. Therefore, changes in DNAm aging from childhood to adolescence may have potential effects on lung function.

To date, only two studies have explored the association of epigenetic aging and lung function. Marioni et al. [16] examined the association of various physical measures with epigenetic aging in over 1000 elderly adults (mean age of 69 ± 0.83 years) in the 1936 Mid-Lothian Birth Cohort, which followed up between three and six years. Lung function, considered as FEV_1_ (forced expiratory volume in one second), showed a statistically weak (*p*-value = 0.05) association with DNAmAge with a small effect size (<1 mL change in FEV_1_ per additional year of epigenetic aging), and epigenetic aging explained only 0.33% of the variance in FEV_1_ decline. In contrast, Rezwan et al. [17] explored the association of lung function in two cohorts, namely the Swiss study of Air Pollution and Lung and heart Disease in Adults (SAPALDIA) and the European Community Respiratory Health Survey (ECRHS) from ALEC (Aging Lungs in European Cohorts) project, at two time points and found that AA is cross-sectionally associated with lower FEV_1_ (forced expiratory volume in one second) and FVC (forced vital capacity) in females at the follow-up time point only. The findings were both statistically significant, and the effect sizes were larger, for FEV_1_: between −5.00 mL and −3.02 mL and for FVC: between −8.06 mL and −4.61 mL, in comparison to the previous study. However, both studies dealt with the association of lung function in comparatively older adults, focusing on lung function decline, and no such work has been undertaken to explore the effect of epigenetic age measures on the lung function development from childhood to adolescence.

Machine learning approaches are increasingly in use to address healthcare problems. However, to date, no study has been conducted to predict lung functions using machine learning approaches. Few studies incorporated machine learning in lung function tests [31] and diseases related to lung function, such as chronic obstructive pulmonary disease (COPD) and asthma [32,33]. Moreover, no work has been done yet to leverage the power of machine learning by utilizing the effect of DNAmAge and AAs on lung function.

As part of the Isle of Wight Birth Cohort (IOWBC), DNAm in peripheral blood and lung function at ages 10 and 18 years were obtained. Therefore, the aim of the study was to explore the efficacy of the use of machine learning regression models in predicting lung function for subjects at 18 years of age using their epidemiological and epigenetic aging data from both 10 and 18 years of age.

## 2. Materials and Methods

### 2.1. Isle of Wight Birth Cohort

The IOWBC is a population birth cohort of 1536 newborns, recruited between 1989 and 1990 [34]. Informed consent for 1456 infants was obtained from the parents, and they were enrolled into the longitudinal study. Participants were followed up at 1 or 2, 4, 10, 18, and 26 years, and peripheral blood samples were collected at birth (neonatal heel prick on Guthrie cards) and at 10, 18, and 26 years.

### 2.2. DNA Extraction and Microarray

DNA was extracted from peripheral blood samples for 326 matched 10 year and 18 year samples. DNAm levels were measured using the Infinium HumanMethylation450 and Methylation EPIC BeadChips from Illumina (Illumina, San Diego, CA, USA) for the 10 year and 18 year old samples, respectively. The CPACOR (Control Probe Adjustment and reduction of global CORrelation) pipeline was used for quality control and pre-processing DNAm data (β values) [35], and batch effect correction was done using ComBat [36].

### 2.3. Measuring Epigenetic Aging

DNAmAge was calculated using the Horvath method, which uses 353 cytosine-phosphate-guanine sites (CpGs) from the Illumina Infinium HumanMethylation450 Beadchip arrays. The missing CpG sites in the EPIC array were imputed during DNAmAge calculation. Age acceleration residuals (AA_res_) were obtained from a linear regression model by regression of DNAmAge on chronological age and further adjusted for blood cell counts to calculate intrinsic epigenetic age acceleration (IEAA). Age acceleration measures were estimated using an online calculator (available at https://dnamage.genetics.ucla.edu/new).

### 2.4. Feature Selection

FEV_1_ and FVC at age 18 were used as the outcome variables. Each subject’s sex, weight, height, hay fever status, asthma status, eczema status, and smoking status at age 18, and FEV_1_ and FVC at age 10 with AA, AA_res_, and IEAA at age 18 were used as features. Mutual information between each feature and the target FEV_1_ and FVC at age 18, respectively, was calculated, and features whose mutual information was > 0.1 were selected. The recursive feature elimination (RFE) method was also undertaken and concurred with the same set of features that were obtained from the mutual information (Table S1). Min-max normalisation was done on selected features before feeding this to the regression model.

### 2.5. Machine Learning Model

Five machine learning regression models: linear, lasso, ridge regression, elastic net, and Bayesian ridge regression, were used to predict FEV_1_ and FVC at age 18. The best subset of features from the feature selection was used, and 10-fold cross-validation was performed along with fine-tuning the hyperparameters using grid search, where applicable. To select the best alpha (hyperparameter that controls the balances between minimizing the residual sum of squares vs. minimizing the sum of squares of coefficients), the models were run for different ranges of alpha, and the best alpha was empirically chosen to build the model. Further, age acceleration changes at 10 and 18 years were added by taking differences between epigenetic age acceleration between two age groups (denoted as: AA_diff_, AA_resdiff_, and IEAA_diff_).

## 3. Results

A total of 326 participants with matched data at 10 and 18 years were analysed. Descriptive statistics are given in Table S2.

### 3.1. Feature Selection by Mutual Information Regression

**Figure 1:**
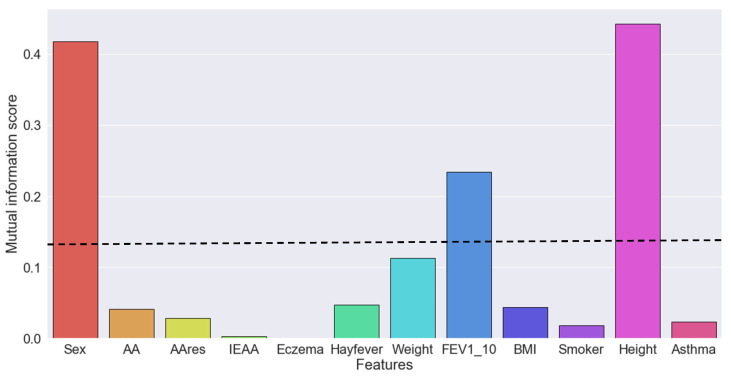
Mutual information score between each feature and the target forced expiratory volume in one second (FEV_1_) at age 18. A mutual information score > 0.1 was used as a threshold for selecting the best features. AA, age acceleration; IEAA, intrinsic epigenetic age acceleration.

For FEV_1_, four features were identified as the most important, namely height, sex, weight, at age 18, and FEV_1_ at age 10 (Figure 1 and Table S3). AA, AA_res_, and IEAA exhibited lower mutual information scores (0.041, 0.028, and 0.003, respectively).

Similarly, for FVC, the same three features (height, sex, and weight) at age 18 and FVC at age 10 were identified as the most important (Figure S1 and Table S2). AA exhibited lower mutual information scores (0.029), and AA_res_ and IEAA had mutual information of zero.

### 3.2. Machine Learning Regression Models for FEV_1_

With the four best features (height, sex, and weight at age 18, and FEV_1_ at age 10) for FEV_1_, all the regression models performed almost similarly after tuning the hyperparameter. However, the ridge regression model (with α = 0.4) worked slightly better (R^2^ = 75.03% ± 7.37% with RMSE = 0.378 ± 0.064) than other methods (Table 1). As expected, based on the mutual information score, adding three age acceleration measures (AA, AA_res_, and IEAA) with these four features did not improve the predictions of FEV_1_ (Table S4).

Changes of AA between the two time points (AA_diff_, AA_resdiff_, and IEAA_diff_) were added with the four predictive features. Although none of the age acceleration differences were found significant during feature selection using mutual information regression, adding AA_diff_ with the other important features showed slight improvement in predicting FEV_1_. The best performer was the ridge regression model (R^2^ = 75.21% ± 7.42% with RMSE = 0.3768 ± 0.0653) (Table 2 and Table S5).

### 3.3. Machine Learning Regression Models for FVC

For FVC, using the four best features (height, sex, and weight at age 18 and FVC at age 10), all the regression models performed with similar efficacy after tuning the hyperparameters. The elastic net regression model (with α = 0.0025) performed slightly better (R^2^ = 75.35% ± 6.88% with RMSE = 0.445 ± 0.064) than the other methods (Table 3). Adding three age acceleration measures (AA, AA_res_, and IEAA) with these four features did not improve the predictions of FVC (Table S6).

While adding changes of AA (AA_diff_, AA_resdiff_, and IEAA_diff_) with the four predictive features, showed almost similar prediction capacity for FVC (Table S7). The best performer was Elastic net regression model (R^2^ = 75.38% ± 6.98% with RMSE = 0.445 ± 0.069) (Table 4).

### 3.4. Effect of Alpha on the Ridge Regression Model

**Figure 2:**
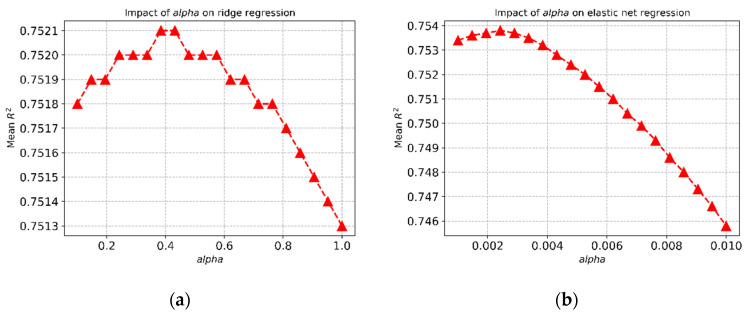
Impact of hyperparameter (α) on (**a**) ridge regression and (**b**) elastic net regression.

The choice of α affects the mean R^2^ values for the regression models. Figure 2a shows how the choice of α affects the mean R^2^ values for ridge regression for the FEV_1_ prediction, and the best R^2^ value was achieved with α = 0.4. Similar behaviour was noticed for the elastic net regression for the FVC prediction (Figure 2b).

## 4. Discussion

Using the data at two time points (10 and 18 years) from IOWBC, we explored whether epigenetic aging can be utilised together with other features for predicting lung function in adolescence using machine learning regression models. Epigenetic age acceleration at 18 years did not contribute to improving the prediction of lung function at 18 years of age. However, using changes in age acceleration between 10 and 18 years improved the prediction of FEV_1_ slightly, despite the fact that the mutual information scores thereof indicated otherwise. Similar improvement, although at an even smaller scale, was observed for FVC.

This is a novel study that examines the effect of epigenetic age acceleration on lung function using supervised machine learning techniques. The previous two studies, examining the association between lung function and epigenetic aging, were performed in an older population and were more focused on lung function decline rather than development. The participants from the Mid-Lothian Birth Cohort study were 70 years at baseline and 76 years at follow-up, and participants from the ALEC project were 37 to 61 years at baseline and 48 to 70 years at follow-up, whereas participants from IOWBC were matched samples at 10 and 18 years.

In this study, changes of epigenetic age acceleration, between 10 years and 18 years, were incorporated with the most informative features from the feature selection technique to develop the best regression models. Previous studies have found height, weight, and sex to be important predictors of lung function [18,37,38]. This study confirms these previous observations and adds lung function at an earlier time point (10 years of age), which confirms the efficacy of machine learning in identifying predictors for lung function. Our study suggests that changes in epigenetic age acceleration between 10 and 18 years can improve the prediction of FEV_1_ and FVC at 18 years of age. Based on the prediction performances of the five selected regression models, it can be postulated that any of these supervised machine learning techniques can be used for lung function prediction.

The fine-tuning of hyperparameters always plays a crucial role in the efficacy of a machine learning technique, and we showed that the choice of the hyperparameter (α) changes the prediction result drastically. Therefore, a grid search was performed for identifying the most optimized parameters for the models to achieve the best prediction performance. This is evident from the higher average R^2^ and lower RMSE values of each regression model. The best models can explain 75.16% and 75.38% of the variance for FEV_1_ and FVC, respectively, through weight, height, sex at 18 years, and lung function at 10 years in conjunction with the changes of epigenetic age acceleration between 10 and 18 years. The RMSE values are also very low for each model (0.3768 ± 0.0653 and 0.4448 ± 0.0690, for FEV_1_ and FVC model, respectively).

Our study has some limitations. Firstly, due to a relatively smaller sample size (*n* = 326), ten-fold cross-validation was used to generate average performance measures of the models rather than using a hold-out test set. However, the cross-validation method performs better to break the bias variance trade-off in small datasets [39]. Furthermore, we note that min-max normalisation on all the data was done before the cross-validation step, whereas, ideally, it is expected that normalization should be done at each step of the cross-validation, learning the normalization only on the training folds and applying it to the test fold. Considering the small dataset and examining that this has virtually no effect on overall performance, this was not followed. Secondly, epigenetic age derived from blood was used rather than lung tissue. However, successful use of epigenetic aging measured from blood is evident in a number of other non-blood related diseases and phenotypes, such as: developmental disorders [40], lung cancer [41], and metabolic syndrome [8]. Additionally, physiological changes, such as hormonal changes during adolescence, were not considered. Moreover, sex-stratified analysis, for lung function, has proven informative in other studies [17,30], and therefore, this could be implemented in this study as well. However, this would further lower the samples size (43.25% female) and may be impractical for this study.

In conclusion, while the full impact of epigenetic age acceleration is still unknown from DNA methylation measures, this study suggests that it can be utilised as one of the potential factors to predict adolescent lung function. It also suggests that the application of machine learning in conjunction with tracking changes in epigenetic age acceleration over the life span can be beneficial to assess lung health in adolescent and have the potential to be extended to adults.

## Figures and Tables

**Table 1:** Results of five regression models predicting FEV_1_ using the best features.

Regression Model	R^2^	RMSE
Linear	74.98 ± 7.45	0.3781 ± 0.06380
Lasso (α = 0.0001)	74.99 ± 7.45	0.3801 ± 0.0519
Ridge (α = 0.4)	75.03 ± 7.37	0.3780 ± 0.0639
Elastic Net (α = 0.001)	75.00 ± 7.41	0.3781 ± 0.0640
Bayesian Ridge	75.01 ± 7.42	0.3780 ± 0.0639

The models were developed using the four best features (height, sex, and weight at age 18 and FEV_1_ at age 10) as predictors of FEV_1_. Here, R^2^ = average goodness-of-fit measure for regression models represented as a percentage and RMSE = average root mean squared error.

**Table 2:** Results of five regression models predicting FEV_1_ using the best features and AA_diff._

Regression Model	R^2^	RMSE
Linear	75.16 ± 7.49	0.3770 ± 0.0652
Lasso (α = 0.0001)	75.16 ± 7.49	0.3770 ± 0.0652
Ridge (α = 0.4)	75.21 ± 7.42	0.3768 ± 0.0653
Elastic Net (α = 0.001)	75.16 ± 7.49	0.3770 ± 0.0653
Bayesian Ridge	75.19 ± 7.46	0.3768 ± 0.0652

The models were developed using the four best features (height, sex, and weight at age 18 and FEV_1_ at age 10) with AA_diff_ as predictors of FEV_1_. Here, AA_diff_ = AA at 18 – AA at 10, R^2^ = average goodness-of-fit measure for regression models represented as a percentage and RMSE = average root mean squared error.

**Table 3:** Results of five regression models predicting FVC using the best features.

Regression Model	R^2^	RMSE
Linear	75.24 ± 7.10	0.4455 ± 0.0692
Lasso (α = 0.0001)	75.25 ± 7.08	0.4456 ± 0.0680
Ridge (α = 0.4)	75.24 ± 7.00	0.4458 ± 0.0673
Elastic Net (α = 0.0025)	75.35 ± 6.88	0.4450 ± 0.0673
Bayesian Ridge	75.25 ± 7.07	0.4456 ± 0.0678

The models were developed using four best features (height, sex, weight at age 18 and FVC at age 10) as predictors of FVC. Here, R^2^ = average goodness-of-fit measure for regression models represented as percentage and RMSE = average root mean squared error.

**Table 4:** Results of five regression models predicting FVC using best features and AA_diff_.

Regression Model	R^2^	RMSE
Linear	75.26 ± 7.14	0.4456 ± 0.0693
Lasso (α = 0.0001)	75.27 ± 7.12	0.4456 ± 0.0692
Ridge (α = 0.4)	75.28 1 7.12	0.4455 ± 0.0691
Elastic Net (α = 0.0025)	75.38 ± 6.98	0.4448 ± 0.0690
Bayesian Ridge	75.28 ± 7.13	0.4455 ± 0.0692

The models were developed using the four best features (height, sex, and weight at age 18 and FVC at age 10) with AA_diff_ as predictors of FVC. Here, AA_diff_ = AA at 18 ‒ AA at 10, R^2^ = average goodness-of-fit measure for regression models represented as a percentage and RMSE = average root mean squared error.

## Supplementary Materials

The following are available online at https://www.mdpi.com/2409-9279/3/4/77/s1: Figure S1: Mutual information score between each feature and the target, which is FVC at age 18. Table S1: Features selected from the recursive feature elimination (RFE) method. Table S2: Summary of the variables IOWBC 10 and 18 year matched samples. Table S3: Mutual information regression scores for predicting FEV_1_ and FVC at 18 years. Table S4: Results of five regression models predicting FEV_1_ using the best features and AAs. Table S5: Results of five regression models predicting FEV_1_ using the best features and AA_resdiff_ and IEAA_diff_. Table S6: Results of five regression models predicting FVC using the best features and AAs. Table S7: Results of five regression models predicting FVC using the best features and AA_resdiff_ and IEAA_diff_.
