# Supplementary material for: Prediction of Lung Function in Adolescence Using Epigenetic Aging: A Machine Learning Approach
Source: Methods Protoc. 2020 Nov 9;3(4):77. doi: 10.3390/mps3040077 (PMC7712054; doi:10.3390/mps3040077)
Supplement: Supplementary file 1 [file mps-03-00077-s001.pdf]

Supplementary materials: Prediction of lung function in adolescence using epigenetic aging: a machine learning approach

Table S1. Features selected from Recursive Feature Elimination (RFE) method.

Features	Ranking
Sex	1
AA	1
AA_{res}	4
IEAA	3
Eczema	7
Hayfever	6
Weight	1
FEV_1 at 10	1
BMI	1
Smoker	2
Height	1
Asthma	5

Here, AA = age acceleration, AA_{res} = age acceleration residual, IEAA = Intrinsic Epigenetic Age Acceleration

Table S2. Summary of the variables IOWBC 10- and 18-year matched samples.

**Features**	**Age 10**	**Age 18**
FEV_1	2.04 ± 0.29	4.151±0.786
Female (%)	43.25	same
Height (cm)	139.07 ± 6.19	171.71 ± 9.39
Weight (kg)	35.22 ± 7.56	69.01 ± 14.25
BMI (kg/m^2)	18.10 ± 2.98	23.32 ± 4.58
Ever asthma	63 (19.33)	94 (28.83)
Eczema	81 (24.85)	61 (18.71)
Hay fever	65 (19.94)	109 (33.46)
AA	19.10 ± 7.98	8.25 ± 8.26
AAres	-0.008 ± 3.99	-0.06 ± 3.78
IEAA	-0.007 ± 3.90	-0.07 ± 3.49

Data are presented as n (%) for categorical and mean ± SD for continuous variables.

Table S3. Mutual information regression scores for predicting FEV1 and FVC at 18 years

Features	FEV1	FVC
Sex	0.4243	0.4015
AA	0.0443	0.0292
AAres	0.0277	0
IEAA	0.0021	0
Eczema	0	0.0314
Hayfever	0.0364	0
Weight	0.114	0.1257
FEV1_10	0.2452	0.2097
BMI	0.0434	0.0046
Smoking	0.0253	0
Height	0.4675	0.5735
Asthma	0.0237	0

Table S4. Results of five regression models predicting FEV_1 using best features and AAs

Added feature	Regression model	R^2	RMSE
AA	Linear	74.91 ± 8.28	0.3782 ± 0.0720
	Lasso (α = 0.0001)	74.90 ± 8.26	0.3784 ± 0.0719
	Ridge (α = 0.4)	74.96 ± 8.16	0.3782 ± 0.0716
	Elastic Net (α = 0.001)	74.91 ± 8.17	0.3785 ± 0.0714
	Bayesian Ridge	74.93 ± 8.23	0.3782 ± 0.0718
AA_{res}	Linear	74.81 ± 7.88	0.3793 ± 0.0683
	Lasso (α = 0.0001)	74.83 ± 7.80	0.3793 ± 0.0682
	Ridge (α = 0.4)	74.87 ± 7.79	0.3790 ± 0.0683
	Elastic Net (α = 0.001)	74.81 ± 7.87	0.3793 ± 0.0680
	Bayesian Ridge	74.84 ± 7.84	0.3791 ± 0.0683
IEAA	Linear	74.77 ± 7.81	0.3796 ± -0.0673
	Lasso (α = 0.0001)	74.77 ± 7.81	0.3796 ± 0.0673
	Ridge (α = 0.4)	74.83 ± 7.72	0.3793 ± 0.0673
	Elastic Net (α = 0.001)	74.79 ± 7.73	0.3796 ± 0.0670
	Bayesian Ridge	74.8 ± 7.77	0.3794 ± 0.0673

The models were developed using best four features (height, sex, weight at age 18 and FEV1 at age 10) with AAs (AA, AA_{res}, and IEAA respectively) as predictors of FEV1. Here, R^2 = average goodness-of-fit measure for regression models represented as percentage and RMSE = average root mean square error

Table S5. Results of five regression models predicting FEV_1 using best features and $AA_{resdiff}$ and $IEAA_{diff}$

Added feature	Regression model	R^2	RMSE
$AA_{resdiff}$	Linear	74.85 ± 7.46	0.3792 ± 0.0644
	Lasso (α = 0.0001)	74.86 ± 7.45	0.3792 ± 0.0644)
	Ridge (α = 0.4)	74.90 ± 7.38	0.3790 ± 0.0646)
	Elastic Net (α = 0.001)	74.90 ± 7.40	0.3789 ± 0.0643)
	Bayesian Ridge	74.88 ± 7.43	0.3791 ± 0.0645
$IEAA_{diff}$	Linear	74.83 ± 7.53	0.3793 ± 0.0647
	Lasso (α = 0.0001)	74.83 ± 7.52	0.3792 ± 0.0646
	Ridge (α = 0.4)	74.88 ± 7.44	0.3791 ± 0.0648
	Elastic Net	74.87 ± 7.46	0.3791 ± 0.0646

	(α = 0.001)		
	Bayesian Ridge	74.86 ± 7.49	0.3791 ± 0.0647)

The models were developed using best four features (height, sex, weight at age 18 and FEV1 at age 10) with $AA_{resdiff}$ and $IEAA_{diff}$ respectively as predictors of FEV1. Here, $AA_{resdiff}$ = AA_{res} at 18 – AA_{res} at 10, $IEAA_{diff}$ = IEAA at 18 – IEAA at 10, R^2 = average goodness-of-fit measure for regression models represented as percentage and RMSE = average root mean square error

Table S6. Results of five regression models predicting FVC using best features and AAs

Added feature	Regression model	R^2	RMSE
AA	Linear	74.85 ± 7.69	0.4485 ± 0.0729
	Lasso (α = 0.0001)	74.87 ± 07.66	0.4484 ± 0.0727
	Ridge (α = 0.4)	74.87 ± 7.60	0.4486 ± 0.0722
	Elastic Net (α = 0.0025)	75.04 ± 7.32	0.4475 ± 0.0711
	Bayesian Ridge	74.87 ± 07.66	0.4485 ± 0.0726
AA_{res}	Linear	75.10 ± 7.30	0.4467 ± 0.0701
	Lasso (α = 0.0001)	75.12 ± 7.28	0.4466 ± 0.0700
	Ridge (α = 0.4)	75.11 ± 7.21	0.4469 ± 0.0693
	Elastic Net (α = 0.0025)	75.24 ± .0701	0.4460 ± 0.0687
	Bayesian Ridge	75.12 ± .0726	0.4467 ± 0.0698
IEAA	Linear	75.01 ± .0741	0.4475 ± 0.0706
	Lasso (α = 0.0001)	75.02 ± .0738	0.4473 ± 0.0704
	Ridge (α = 0.4)	75.02 ± .0732	0.4476 ± 0.0698
	Elastic Net (α = 0.0025)	75.18 ± .0708	0.4464 ± 0.0689
	Bayesian Ridge	75.02 ± .0737	0.4474 ± 0.0703

The models were developed using best four features (height, sex, weight at age 18 and FVC at age 10) with AAs (AA, AA_{res}, and IEAA respectively) as predictors of FVC. Here, R^2 = average goodness-of-fit measure for regression models represented as percentage and RMSE = average root mean square error

Table S7. Results of five regression models predicting FVC using best features and $AA_{resdiff}$ and $IEAA_{diff}$

Added feature	Regression model	R^2	RMSE
$AA_{resdiff}$	Linear	75.13 ± .0703	0.4467 ± 0.0668
	Lasso (α = 0.0001)	75.14 ± .0703	0.4466 ± 0.0669
	Ridge (α = 0.4)	75.13 ± .0696	0.4469 ± 0.0662
	Elastic Net (α = 0.0025)	75.23 ± .0689	0.4461 ± 0.0669
	Bayesian Ridge	75.14 ± .0700	0.4467 ± 0.0666
$IEAA_{diff}$	Linear	75.26 ± .0685	0.4458 ± 0.0659
	Lasso (α = 0.0001)	75.27 ± .0685	0.4457 ± 0.0660
	Ridge (α = 0.4)	75.25 ± .0677	0.4460 ± 0.0653
	Elastic Net (α = 0.0025)	75.32 ± .0677	0.4455 ± 0.0660
	Bayesian Ridge	75.27 ± .0682	0.4457 ± 0.0656

The models were developed using best four features (height, sex, weight at age 18 and FVC at age 10) with $AA_{resdiff}$ and $IEAA_{diff}$ respectively as predictors of FVC. Here, $AA_{resdiff}$ = AA_{res} at 18 – AA_{res} at 10, $IEAA_{diff}$ = IEAA at 18 – IEAA at 10, R^2 = average goodness-of-fit measure for regression models represented as percentage and RMSE = average root mean square error

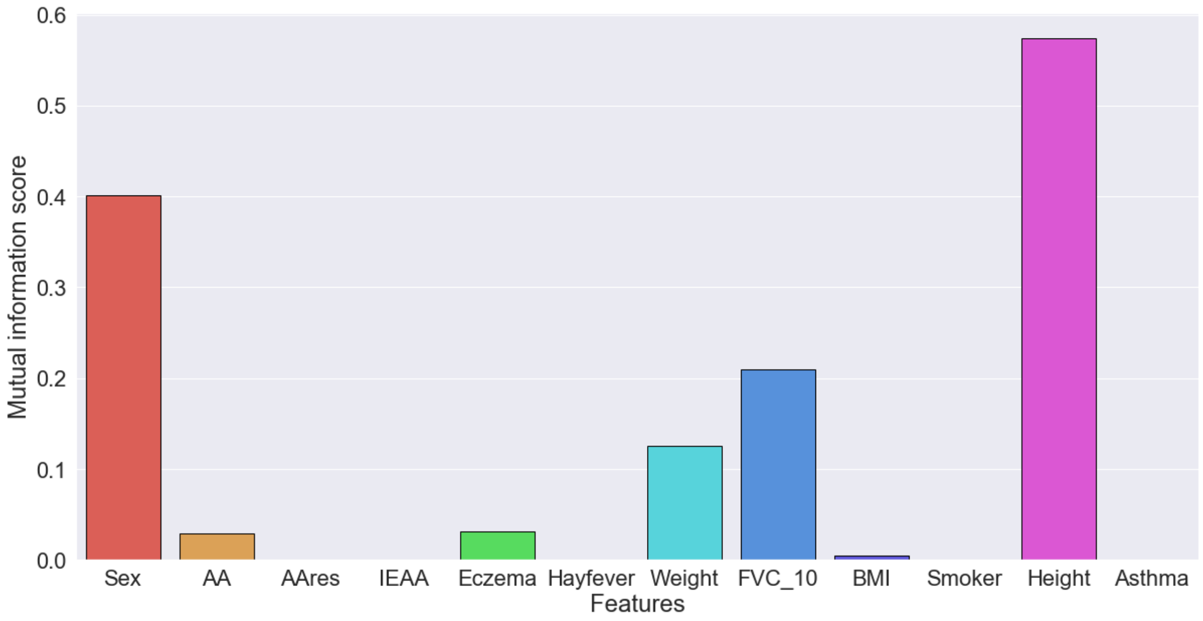

Figure S1. Mutual information score between each feature and the target which is FVC at age 18. Association of height and gender with FVC is higher than any of the other features.
